# Impact of A Cargo-Less Liposomal Formulation on Dietary Obesity-Related Metabolic Disorders in Mice

**DOI:** 10.3390/ijms21207640

**Published:** 2020-10-15

**Authors:** Varsha Komalla, Behjat Sheikholeslami, Gerard Li, Bishwajit Bokshi, Yik Lung Chan, Alison Ung, Brian Gregory Oliver, Hui Chen, Mehra Haghi

**Affiliations:** 1 Graduate School of Health, University of Technology Sydney, Chippendale, NSW 2008, Australia; varsha.komalla@student.uts.edu.au (V.K.); behjat.sheikholeslami@student.uts.edu.au (B.S.); bishwajit.bokshi@student.uts.edu.au (B.B.); 2 Woolcock Institute of Medical Research, The University of Sydney, Glebe, NSW 2037, Australia; jeremy.yl.chan@gmail.com (Y.L.C.); brian.oliver@uts.edu.au (B.G.O.); 3 School of Life Sciences, University of Technology Sydney, Ultimo, NSW 2007, Australia; gerard.E.li@student.uts.edu.au (G.L.); hui.chen-1@uts.edu.au (H.C.); 4 School of Mathematical and Physical Sciences, University of Technology Sydney, Ultimo, NSW 2007, Australia; alison.ung@uts.edu.au

**Keywords:** inflammation, obesity, 1,2-dioleoyl-sn-glycero-3-phosphocholine (DOPC) liposomes, high fat diet

## Abstract

Current therapeutic options for obesity often require pharmacological intervention with dietary restrictions. Obesity is associated with underlying inflammation due to increased tissue macrophage infiltration, and recent evidence shows that inflammation can drive obesity, creating a feed forward mechanism. Therefore, targeting obesity-induced macrophage infiltration may be an effective way of treating obesity. Here, we developed cargo-less liposomes (UTS-001) using 1,2-dioleoyl-sn-glycero-3-phosphocholine, DOPC (synthetic phosphatidylcholine) as a single-agent to manage weight gain and related glucose disorders due to high fat diet (HFD) consumption in mice. UTS-001 displayed potent immunomodulatory properties, including reducing resident macrophage number in both fat and liver, downregulating liver markers involved in gluconeogenesis, and increasing marker involved in thermogenesis. As a result, UTS-001 significantly enhanced systemic glucose tolerance in vivo and insulin-stimulated cellular glucose uptake in vitro, as well as reducing fat accumulation upon ad libitum HFD consumption in mice. UTS-001 targets tissue residence macrophages to suppress tissue inflammation during HFD-induced obesity, resulting in improved weight control and glucose metabolism. Thus, UTS-001 represents a promising therapeutic strategy for body weight management and glycaemic control.

## 1. Introduction

A third of the global population is affected by obesity and the population of obese people has been doubled since the 1980s [1]. Obesity is a complex multifactorial disease characterized by excess fat deposits causing dyslipidaemia and is strongly associated with insulin resistance leading to glucose intolerance and eventually type 2 diabetes [2,3]. Current obesity management depends on lifestyle modification alone or in combination with either pharmacological treatment or bariatric surgery according to the severity of obesity and co-morbidities [4]. Common lifestyle modifications achieve little effects in weight management due to low adherence [5]. Anti-obesity drugs are associated with serious side effects (psycho-stimulatory, depression, addiction, gastrointestinal effects) [6,7,8,9]. Bariatric surgery is the only effective therapy used in patients who are morbidly obese, but this carries several side effects such as osteoporosis and malnutrition in the long term [10]. Thus, there is a need for developing new effective therapeutics with fewer side effects to manage body weight and related metabolic disorders.

Obesity is exemplified by chronic systemic low-grade inflammation, marked by the recruitment of monocytes into metabolic organs, which then form resident macrophages, such as adipose tissue macrophages (ATM) or Kupffer cells in the liver [11]. These resident macrophages release pro-inflammatory cytokines, such as tumor necrosis factor-alpha (TNF-α) and interleukin-6 (IL-6) [12], which act locally and systemically to impair insulin sensitivity and related glucose uptake. TNF-α also causes pre-adipocytes to differentiate into macrophage-like cells that produce more pro-inflammatory cytokines [13]. Thus, reducing the recruitment of macrophages has been shown to protect mice from obesity-induced insulin resistance [14]. Moreover, inhibiting pro-inflammatory cytokine release has been shown to increase lipolysis, resulting in fat loss and improved insulin sensitivity in obese mice [15,16]. However anti-TNF-α treatment failed in the clinical trial, suggesting a new anti-inflammatory approach needs to target macrophages instead of individual pro-inflammatory cytokines to effectively manage body weight and metabolic disorders in obesity.

Recent studies have demonstrated the efficacy of a mixture of natural phosphatidylcholines (PCs) in diet-induced obesity and hyperlipidemia in vivo [17,18], with another two studies reporting antidiabetic effects and reducing liver mitochondrial injury following dilinoleoylphosphatidylcholine (DLPC) administration [19,20]. Furthermore, PCs in the form of liposome are currently used in several registered formulations on the market, but no studies to date have investigated the therapeutic effect of the cargo-less liposomal formulation of PCs.

Natural phospholipids are available as a heterogeneous mixture of PCs with different fatty acyl chains. Due to the presence of multiple unsaturated fatty acids, natural PCs are generally unstable [21]. In the present study, a pure, synthetic PC, 1,2-Dioleoyl-sn-glycero-3-phosphocholine (DOPC) has been formulated as liposomes without any active therapeutic loaded inside (cargo less liposome-UTS-001). In our previous studies, UTS-001 significantly reduced IL-6 release following TNF-α stimulation in airway epithelial cells in vitro and reduced airway inflammation in a mouse model of allergic asthma [22]. Similarly, metabolic disorders are caused by systemic low-grade inflammation with pro-inflammatory cytokines playing a significant role in disease development. The anti-inflammatory effect of UTS-001 may target resident macrophages in the obese to exert therapeutic efficacy by reducing fat accumulation and improving glucose disorders.

Therefore, in the current study, mice on a high-fat diet (HFD) were intraperitoneally injected with the UTS-001 to examine the efficacy of this liposomal formulation on reducing resident macrophage, related inflammatory parameters, and fat accumulation.

## 2. Results

### 2.1. In Vivo Studies

#### 2.1.1. Anthropometric and Metabolic Parameters

To model obesity, mice were fed a HFD (20 KJ/g, 43% fat) for 6 weeks. Control mice were fed standard rodent chow (12 KJ/g, 14% fat). From the 7th week, 2 doses of UTS-001 (0.6 mg and 6 mg) were administered once daily to HFD-fed mice for four weeks while the maintenance diet remained unchanged. As shown in Table 1, at week 0, the mean body weight of HFD-fed mice was the same as the chow-fed mice. At the endpoint, no significant difference was observed in body weight among chow-fed mice regardless of UTS-001 dose, suggesting no toxicity. Interestingly, the final body weight of the HFD-UTS-001 (6 mg) group was 8.9% lower compared to that of the HFD-PBS group (*p* < 0.05). The final body weight of HFD fed mice receiving UTS-001 (0.6 mg) did not show any significant change compared to HFD-PBS mice. There was a significant increase (*p* < 0.0001) in calorie intake of HFD-fed groups compared to Chow-fed groups. Furthermore, the HFD mice treated with UTS-001 (0.6 mg) had a significant increase (*p* < 0.05) in calorie intake compared to HFD-PBS mice. this effect was not seen in UTS-001 (6 mg) treated HFD mice.

HFD-PBS group demonstrated significantly greater retroperitoneal fat content, liver weight and testicular fat levels compared to the Chow-PBS group (*p* < 0.0001 for all, Table 1). Furthermore, there was no significant difference in fat mass between chow mice receiving UTS-001 or PBS while a significant decrease in liver weight (*p* < 0.05), retroperitoneal (Rp) fat (*p* < 0.001), and testicular fat mass (*p* < 0.0001) were observed between HFD-UTS-001 (6 mg) group and HFD-PBS group. No significance in liver weight% was observed between the groups. With respect to Rp fat % and testicular fat %, UTS-001 (6 mg) treatment significantly reduced these parameters when compared to HFD-PBS group (*p* < 0.01 for Rp fat % and *p* < 0.0001 for Testicular fat %). Among all the other organs (Tibia, heart, spleen) only heart weight % was significantly reduced in all HFD-fed groups compared to Chow-PBS.

As demonstrated in Table 2, serum triglycerides (TG) level in HFD fed mice were significantly higher compared to the Chow-PBS group (HFD-PBS, *p* < 0.05) which were not affected by UTS-001 (6 mg) treatment (Table 2). Serum free fatty acid (FFA) level was 15% higher in HFD-PBS group, which was normalized in HFD-UTS-001 (6 mg), although without statistical significance (Table 2).

**Figure 1:**
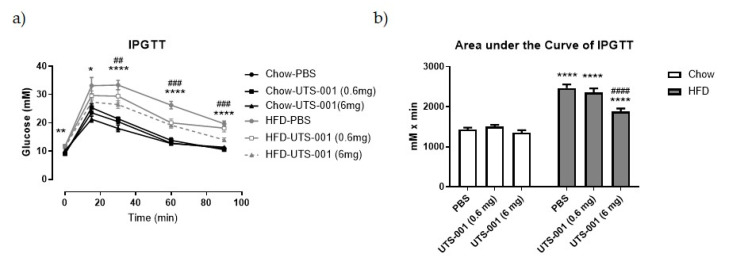
(**a**) An intraperitoneal glucose tolerance test (IPGTT, glucose 2 g/kg) after 4 weeks of treatment. (**b**) Area under the curve (AUC) of the IPGTT in (**a**). Data are expressed as mean ± S.E.M, *n* = 7–12. Data were analysed using two-way ANOVA followed by post hoc Fischer’s LSD tests. In (**a**), ** *p* < 0.01, Chow-PBS vs. HFD-PBS at o min; * *p* < 0.05, Chow-PBS vs. HFD-PBS at 15 min; **** *p* < 0.0001, Chow-PBS vs. HFD-PBS at 30, 60, 90 min, ### *p* < 0.001, HFD-PBS vs. HFD-UTS-001 at 60, 90 min and ## *p* < 0.01, HFD-PBS vs. HFD-UTS-001 at 30 min. in (**b**) **** *p* < 0.0001 vs. Chow-PBS; #### *p* < 0.0001 vs. HFD-PBS group.

Fasting blood glucose concentrations in HFD-PBS group were significantly increased (*p* < 0.01 vs. Chow-PBS, Table 2), which was not changed by UTS-001 (0.6 mg and 6 mg) treatment. There was no significant difference in insulin levels among any groups. Homeostatic Model Assessment of Insulin Resistance (HOMA-IR), an indicator of insulin resistance, was significantly increased (*p* < 0.0001) in HFD-PBS group compared to the Chow-PBS group, which was not changed by UTS-001 treatment. As shown in Figure 1a, during intraperitoneal glucose tolerance test (IPGTT) blood glucose level was significantly increased in the HFD-PBS group at 15, 30, 60 and 90 min as expected (*p* < 0.0001 for 30, 60, 90 min; *p* < 0.05 for 15 min and *p* < 0.01 for 0 min vs. Chow-PBS). HFD mice receiving UTS-001 (6 mg) treatment had significantly lowered blood glucose levels in comparison to the HFD-PBS group (*p* < 0.001 for 60 and 90 min, *p* < 0.01 for 15 min). The area under the curve (AUC) was significantly higher in the HFD-PBS groups (*p* < 0.0001 vs. Chow-PBS). Interestingly, in the HFD-UTS-001 (6 mg) group, AUC has been decreased significantly (*p* < 0.0001 vs. HFD-PBS, Figure 1b), suggesting improved glucose tolerance by UTS-001 (6 mg) treatment.

Thus, four weeks of UTS-001 (6 mg) treatment reduced body weight gain and fat mass with no effect on plasma TG in HFD-fed mice. Although insulin resistance was not improved, systemic glucose metabolism has been significantly improved upon high glucose challenge. To investigate if the components of UTS-001 (DOPC and cholesterol mixture) affect these parameters, a second cohort of animals was used. There was no significant weight change, nor significant improvement in glucose tolerance following treatment with non-liposomal DOPC and cholesterol mixture, whilst a small but significant reduction was observed with DOPC liposomes (Supplementary Figure S1), the effect of which was not as potent as UTS-001.

#### 2.1.2. Inflammatory Markers

**Figure 2:**
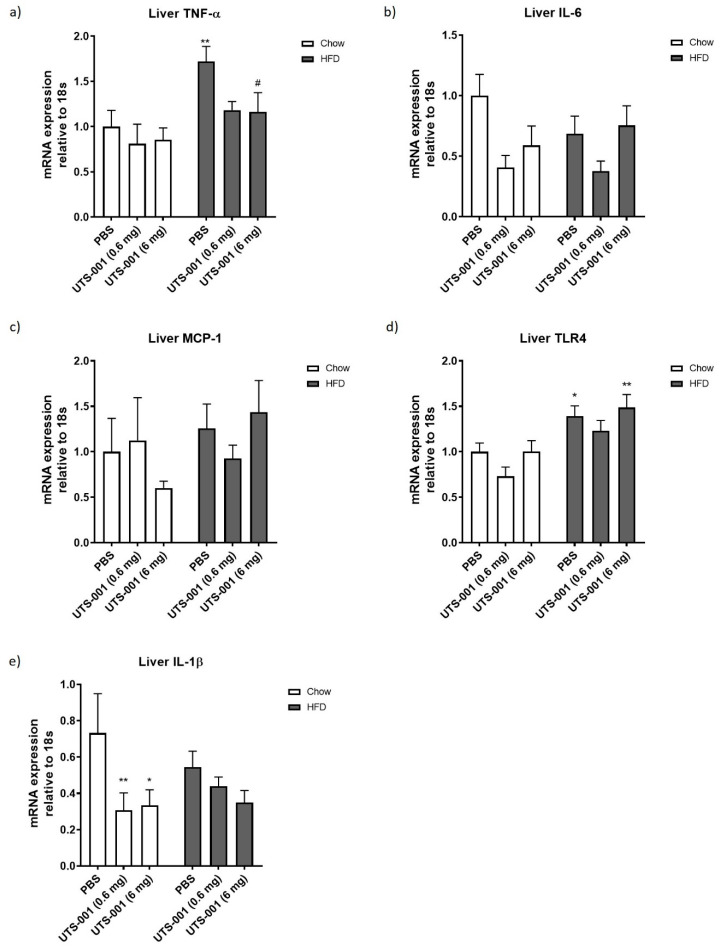

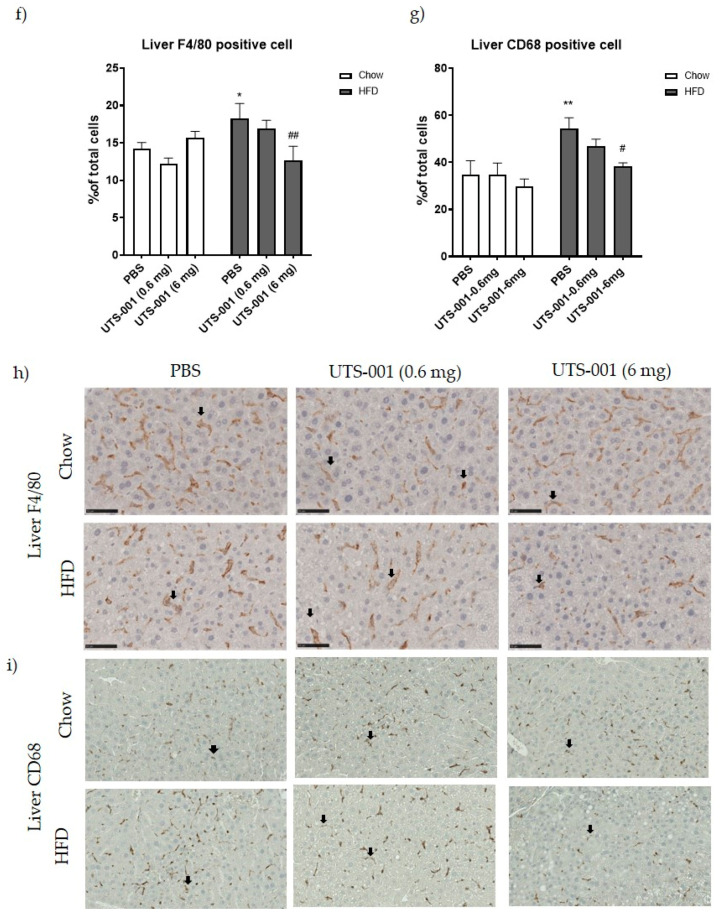
Liver mRNA expression of inflammatory markers (**a**) TNF-α, (**b**) IL-6, (**c**) MCP-1, (**d**) TLR-4, (**e**) IL-1β. Percentage of macrophage number as identified by (**f**) F4/80 and (**g**) CD68 immunohistochemistry staining and (**h**) and (**i**) respective representative images were taken at 10× magnification. Positive staining indicated by arrows in h and i. Data are represented as mean ± S.E.M and were analysed using two-way ANOVA followed by post hoc Fischer’s LSD test, *n* = 5–9. * *p* < 0.05 vs. Chow-PBS; ** *p* < 0.01 vs. Chow-PBS; # *p* < 0.05 vs. HFD-PBS; ## *p* < 0.01 vs. HFD-PBS.

In the liver, HFD consumption alone significantly up-regulated TNF-α and toll-like receptor-4 (TLR4) mRNA expression (TNF-α, *p* < 0.01 HFD-PBS vs. Chow-PBS, Figure 2a; TLR4, *p* < 0.05 HFD-PBS vs. Chow-PBS, Figure 2d). No significant changes in mRNA expression of liver IL-6 (Figure 2b) and monocyte chemoattractant protein-1 (MCP-1) (Figure 2c) have been observed among the groups. Interleukin 1-beta (IL-1β) mRNA expression was decreased in UTS-001 treated Chow-fed mice (*p* < 0.01 for 0.6 mg and *p* < 0.05 for 6 mg vs. Chow-PBS group) (Figure 2e).

Macrophages number in the liver was assessed using F4/80 and CD68 staining. Liver F4/80 positive cells have been increased (*p* < 0.05) in the HFD-PBS group (Figure 2f), which has been markedly decreased in the HFD-UTS-001 (6 mg) group (*p* < 0.01 vs. HFD-PBS). CD68 positive cells were also markedly increased in HFD-PBS group compared to Chow-PBS group (*p* < 0.01), which was significantly decreased in the HFD-UTS-001 (6 mg) group (*p* < 0.05 vs. HFD-PBS, Figure 2g).

**Figure 3:**
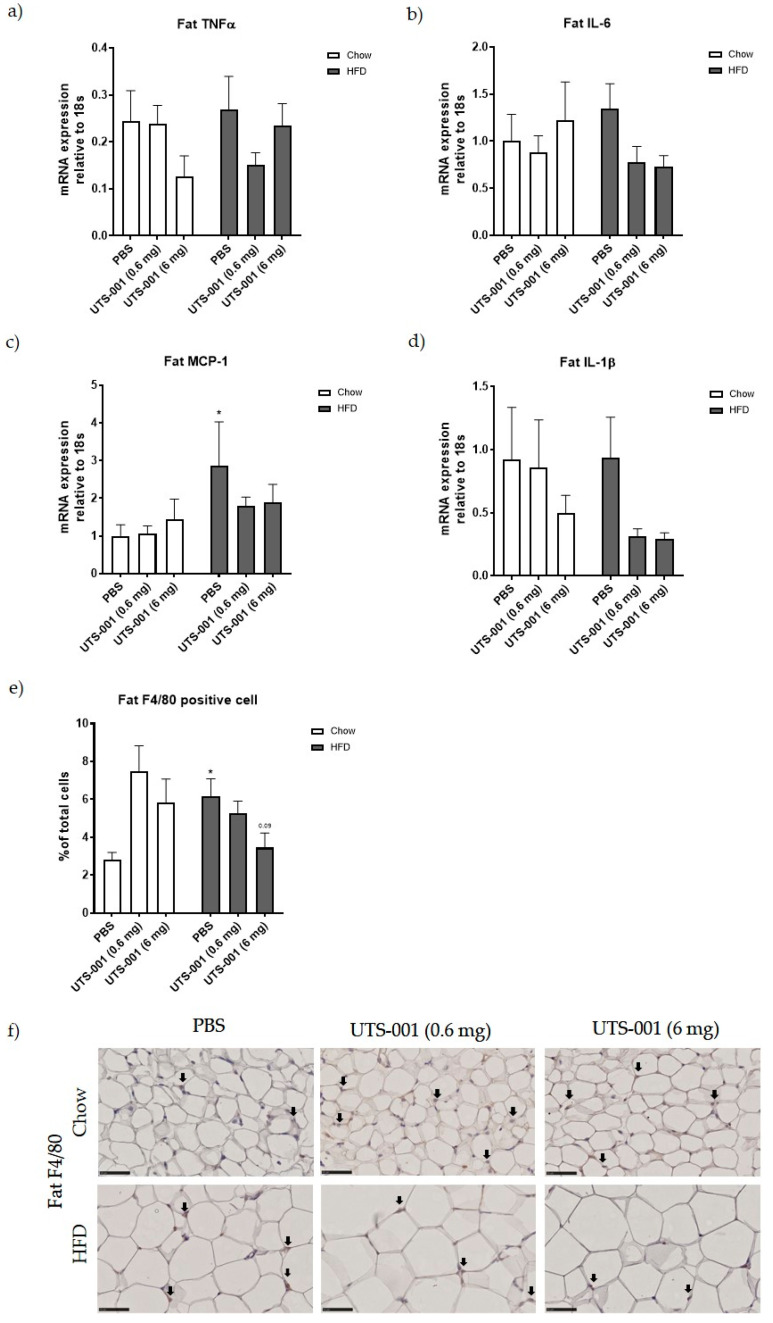
Fat mRNA expression of (**a**) TNF-α, (**b**) IL-6, (**c**) MCP-1, (**d**) IL-1β and (**e**) Percentage of macrophage number in the fat as identified by F4/80 immunohistochemistry and (**f**) Respective representative images for the stain taken at 10× magnification. Positive staining indicated by arrows in figure f. Data are represented as mean ± S.E.M and were analysed using two-way ANOVA followed by post hoc Fisher’s LSD test, *n* = 6–9. * *p* < 0.05 vs. Chow-PBS.

In the fat tissue, no significant changes in TNF-α (Figure 3a) and IL-6 (Figure 3b) mRNA expression were observed among all groups. Fat MCP-1 (Figure 3c) mRNA expression was significantly increased in HFD-PBS group compared to Chow-PBS (*p* < 0.05), which was lower in UTS-001 treated groups albeit with no statistical significance. Fat IL-1β mRNA expression was not significantly different among all groups, although the levels were reduced by more than 50% in HFD-fed mice after UTS-001 treatment (Figure 3d). F4/80 positive cell number in fat tissue in the HFD-PBS group (Figure 3b) was significantly higher than the Chow-PBS (*p* < 0.05). In the HFD-UTS-001 (6 mg) group, F4/80 positive cells were decreased by nearly 50% but this change was not statistically significant (*p* = 0.09 vs. HFD-PBS).

#### 2.1.3. mRNA Expression of Metabolic Markers in Livertitle

**Figure 4:**
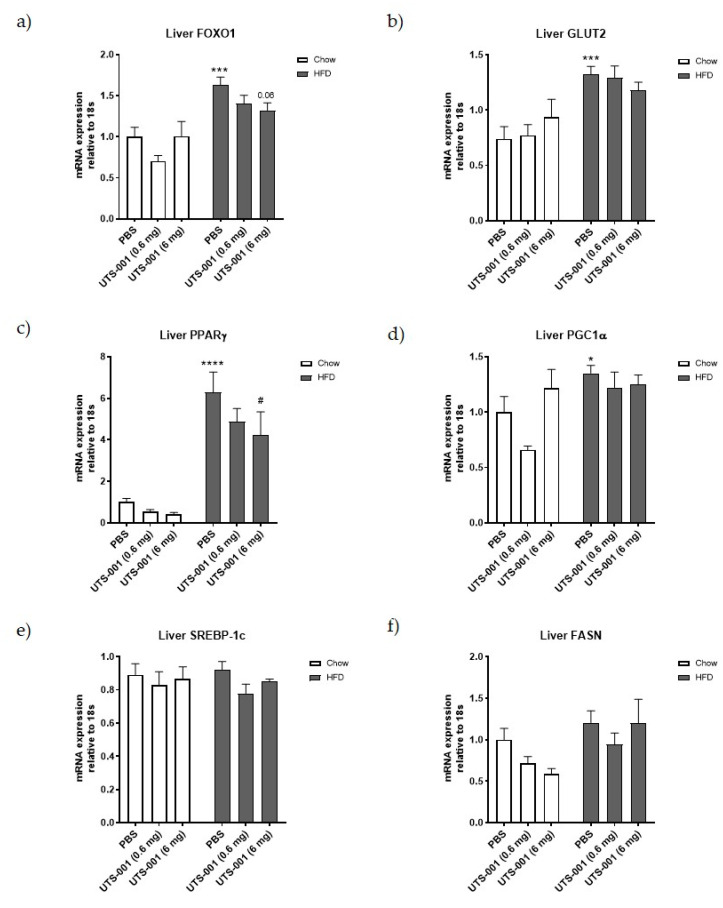

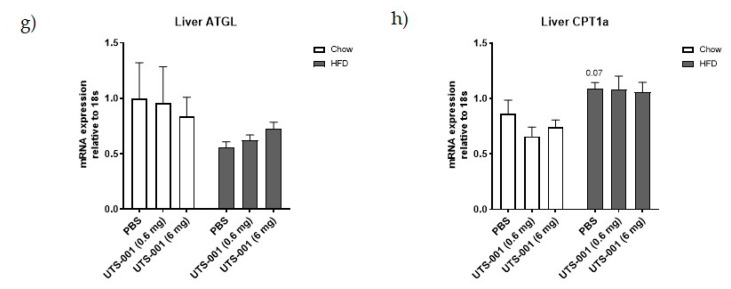
mRNA expression of liver glucose and lipids markers (**a**) FOXO-1, (**b**) GLUT-2, (**c**) PPARγ, (**d**) PGC-1α, (**e**) SREBP-1c, (**f**) FASN, (**g**) ATGL, (**h**) CPT-1a. Results are expressed as mean ± S.E.M, *n* = 7–9. Data were analyzed by two-way ANOVA followed by post hoc Fischer’s test. * *p* < 0.05 vs. Chow-PBS; *** *p* < 0.001 vs. Chow-PBS; **** *p* < 0.0001 vs. Chow-PBS, *p* = 0.06 vs. HFD-PBS, # *p* < 0.0001 vs. HFD-PBS.

As shown in Figure 4, a significant increase in mRNA expression of glucose metabolic markers, including forkhead box protein O1 (FOXO1) (*p* < 0.001, 4a), glucose transporter 2 (GLUT2) (*p* < 0.001, 4b), peroxisome proliferator-activated receptor (PPAR)γ (*p* < 0.0001, 4c), and peroxisome proliferator-activated receptor gamma coactivator 1-alpha (PGC-1α) (*p* < 0.05, 4d) levels are seen in the liver of the HFD-PBS group compared to the Chow-PBS group. A decrease in the expression of PPARγ (*p* < 0.05 vs. HFD-PBS) in the HFD-UTS-001 (6 mg) group was noted. Furthermore, treatment with UTS-001 (6 mg) in the HFD group resulted in a reduction of FOXO-1 by 19% (*p* = 0.06) and GLUT2 by 15% compared to HFD-PBS, which was not statistically significant.

No significant difference was observed in liver lipid metabolic markers, including Sterol regulatory element-binding protein 1 (SREBP-1c), fatty acid synthase (FASN), adipose triglyceride lipase (ATGL), and carnitine palmitoyltransferase 1a (CPT-1a) mRNA expression among all the groups.

#### 2.1.4. mRNA Expression of Metabolic Markers in the Fat

**Figure 5:**
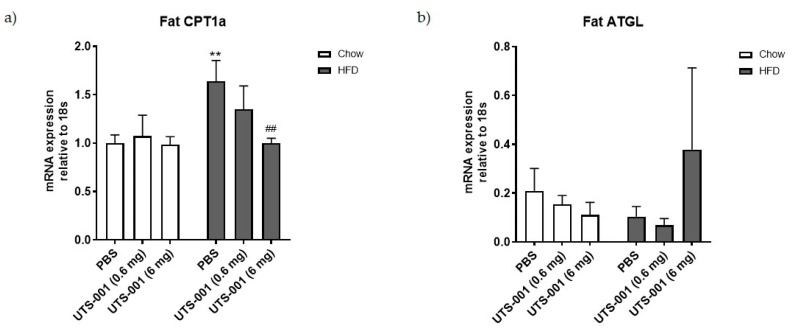

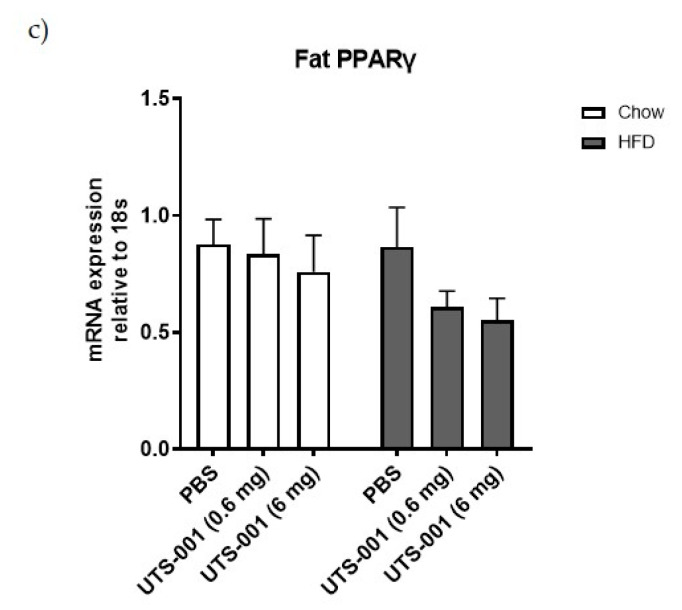
mRNA expression of (**a**) CPT-1a; (**b**) ATGL; (**c**) PPARγ in the fat. Results are expressed as mean ± S.E.M, *n* = 7–9. Data were analyzed by two-way ANOVA followed by post hoc Fisher’s test. ** *p* < 0.01 vs. Chow-PBS; ## *p* < 0.01 vs. HFD-PBS.

In the fat tissue, a significant increase in mRNA expression of CPT-1a was observed in the HFD-PBS group compared to the Chow-PBS group (*p* < 0.01 Figure 5a), which was normalised by UTS-001 (6 mg) treatment (*p* < 0.01, HFD-UTS-001 (6 mg) vs. HFD-PBS). Furthermore, no significant difference in fat ATGL mRNA expression was observed, although the level was more than tripled in the HFD-UTS-001 (6 mg) group compared with the HFD-PBS group (Figure 5b) and no significant difference in PPARγ mRNA expression was observed among all groups (5c).

#### 2.1.5. mRNA Expression of Thermogenesis Markers in Brown Fat

**Figure 6:**
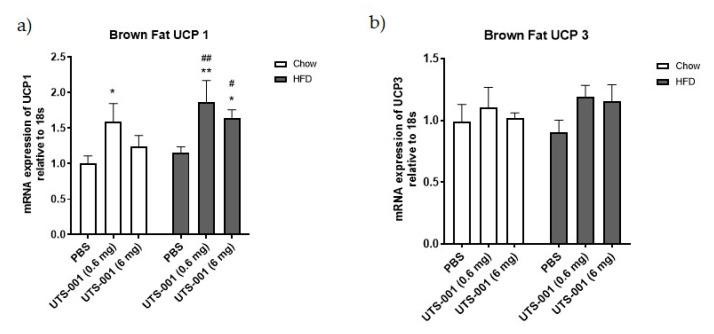
mRNA expression of (**a**) UCP-1; (**b**) UCP-3 in the brown fat. Results are expressed as mean ± S.E.M, *n* = 7–9. Data is analyzed by two-way ANOVA followed by post hoc Fischer’s LSD test. * *p* < 0.05 vs. Chow-PBS; ** *p* < 0.01 vs. Chow-PBS; # *p* < 0.05 vs. HFD-PBS; ## *p* < 0.01 vs. HFD-PBS.

Chow-fed mice receiving UTS-001 (0.6 mg) had shown a significant rise (*p* < 0.05) in Uncoupling protein-1 (UCP-1) levels compared to chow-PBS mice. UCP-1 mRNA expression level was also significantly increased in HFD fed mice treated with UTS-001 (0.6 mg) (*p* < 0.01 vs. chow-PBS and *p* < 0.01 vs. HFD-PBS group) and UTS-001 (6 mg) (*p* < 0.05 vs. chow-PBS and *p* < 0.05 vs. HFD-PBS groups) (Figure 6a). Moreover, there were no changes in UCP-3 levels among the groups (Figure 6b).

### 2.2. Effect of UTS-001 on Uptake of 2-(N-(7-Nitrobenz-2-oxa-1, 3-Diazol-4-yl) Amino)-2 Deoxyglucose (2-NBDG), Number and Diameter of Adipocytes In Vitro

**Figure 7:**
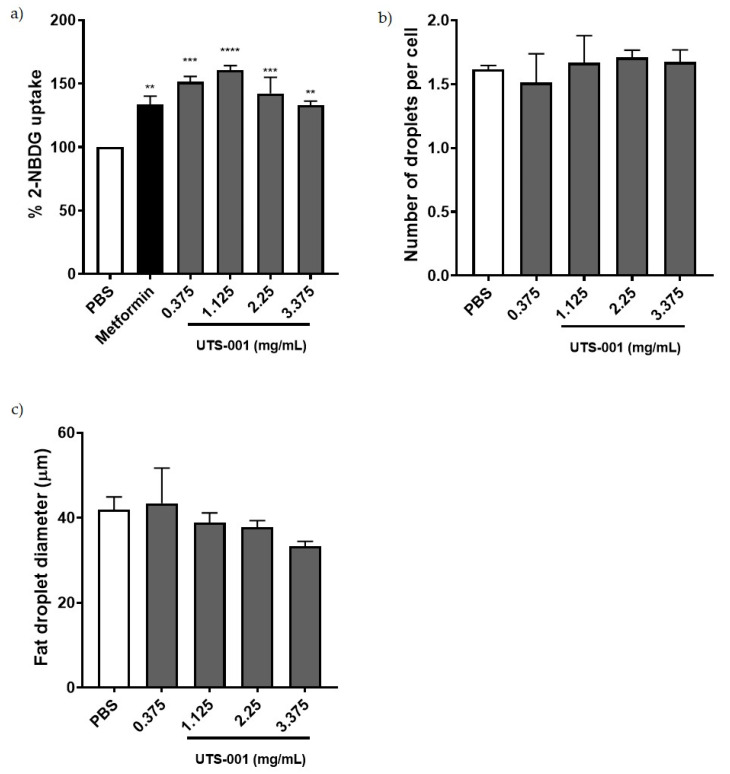
(**a**) Insulin-mediated glucose uptake in mature adipocytes pre-treated with PBS, Metformin, and UTS-001. (**b**) number of fat droplets;(**c**) diameter of fat droplets treated with PBS, and UTS-001. Results are expressed as mean ± S.E.M. Data were analysed by one-way ANOVA followed by post hoc Fisher’s LSD tests. ** *p* < 0.01 vs. PBS; *** *p* < 0.001 vs. PBS; **** *p* < 0.0001 vs. PBS; *n* = 3.

We performed a glucose uptake assay in differentiated mature adipocytes from the 3T3L cell line. As shown in Figure 7a, the fluorescently labeled glucose analogue, 2-NBDG was significantly increased by the first line anti-diabetic drug metformin (*p* < 0.01) as a positive control. All the concentrations of UTS-001 (*p* < 0.01 for 3.375 mg/mL, *p* < 0.001 for 0.375 and 2.25 mg/mL, *p* < 0.0001 for 1.125 mg/mL of lipids) have achieved similar effects as metformin to increase glucose uptake in matured adipocytes. As shown in Figure 7b,c, no significant changes were observed in the number and diameter of adipocytes among groups after treatment with UTS-001, although there was a trend of decreasing adipocyte diameter with the increase in UTS-001 dose.

## 3. Discussion

Obesity is a low-grade systemic inflammatory disease induced by increased and overactivation of tissue resident macrophages, hence targeting residence macrophages may assist weight management and related metabolic disorders. In the current study, we have developed a liposomal formulation of DOPC (UTS-001) that is able to reduce residence macrophage number and inflammation. UTS-001(6 mg) treatment protected mice against weight gain and fat accumulation with ad libitum consumption of a HFD. Furthermore, UTS-001(6 mg) treatment decreased glucose intolerance in obese mice representing pre-diabetes conditions in humans [23]. Furthermore, an increase in thermogenesis marker, reflected by UCP-1 levels in brown fat, may serve as an additional mechanism behind the fat loss effect.

HFD consumption resulted in an increase in body weight, fat mass, and liver weight consistent with our previous study using the same model [24]. Daily injection of UTS-001 (6 mg) can limit such obesogenic effects without affecting daily caloric intake. Clinical studies have demonstrated that only a 5–10% weight loss can result in a significant improvement in obesity-related metabolic disorders [25]. In the current study, 8.9% of weight loss was achieved following four weeks of daily administration of UTS-001, resulting in significantly improved systemic glucose uptake during IPGTT, the gold standard method to diagnose diabetes. This effect was only apparent in the high dose (6 mg) group. Glucose uptake improvement by UTS-001 was further confirmed in the in vitro studies where we observed an increase in the insulin-dependent uptake of 2-NBDG in mature adipocytes. This is in line with the efficacy of Caulerpa lentillifera extract as an antidiabetic drug shown by Sharma et al. [26]. Adipocytes play vital roles in regulating energy balance and glucose homeostasis, where enlarged adipocyte size is associated with insulin resistance [27]. Although the HOMA results did not suggest a systemic improvement in insulin resistance, upon a high level of glucose challenge, tissue glucose uptake was improved. Glucose homeostasis is regulated by insulin/AKT and AMPK pathways in insulin-response tissues, which promotes GLUT4 translocation to the cell membrane for postprandial glucose uptake. Improved systemic glucose clearance during IPGTT in this study could be through the modulation of this insulin/AKT and AMPK pathways when insulin secretion is increased in response to a high level of circulating glucose challenge [28,29,30] indirectly supported by our in vitro study. Furthermore, improved glucose uptake in HFD-fed mice in this study could also be explained by the fact that phospholipase A2 (PLA2) is normally elevated in inflamed organs [31]. The breakdown of DOPC liposomes by PLA2 is known to release oleic acid and unsaturated 18:1 lysophosphatidylcholine (LPC) [32]. This LPC species acts via GPR119, a key receptor that regulates the secretion of insulin from β cells of pancreatic islets [33], which may explain the reduced blood glucose level following IPGTT. The impact of UTS-001 on β cell function requires further investigation, which is beyond the scope of this study. Together, the results from our in vivo and in vitro studies support a novel role for UTS-001 in improving glucose uptake in mice.

During obesity, there is an increased number of macrophages infiltration in adipose tissue and liver [34,35]. Increased Lipid influx and accumulation in those organs cause the activation of Kupffer cells (liver resident macrophages) and monocyte-derived macrophages. Moreover, chronic hepatic inflammation leads to fatty liver disease and cirrhosis [35]. In the current study, macrophages were increased in both liver and fat tissue of HFD-PBS group, which were decreased with reduced inflammatory responses by UTS-001 treatment in both organs. TNF-α produced by tissue-resident macrophages causes metabolic syndrome and insulin resistance [36,37]. In humans, addressing this pathophysiological mechanism has been challenging. Corticosteroids, the most effective anti-inflammatory medications, cause obesity and diabetes at high doses (via gluconeogenesis and thereafter fat re-distribution), and the recent ASCEND (A Study of Cardiovascular Events in Diabetes) trial of the non-steroidal anti-inflammatory drug (NSAID), aspirin in diabetes found the risk profile to outweigh any benefit [38]. Anti-TNF therapies used clinically for immune/autoimmune diseases have failed the clinical trial to manage body weight in obese people [39], suggesting that targeting TNF alone is not effective. In the current study, we observed broader efficacy by UTS-001 treatment which is evident by reduced fat mass, body weight and improved glucose metabolism in the face of reduced tissue macrophage numbers and inflammatory response consistent with the hypothesis of the effectiveness of targeting macrophages to improve obesity-related metabolic disorder [40]. MCP-1 is a key chemokine involved in the infiltration of monocytes and elevated levels of this chemokine is witnessed in white adipose tissue which is consistent with the findings in the current study [41]. However, there is no change after UTS-001 (6 mg) treatment indicating that a decrease in monocyte recruitment may not be the mechanism through which UTS-001 (6 mg) reduced macrophage number. The migration of macrophages out of the resident tissue after engulfing UTS-001 may be increased, which requires further investigation to confirm. In chow-fed mice, there was an apparent (non-significant) increase in the number of adipose tissue macrophages, whereas significantly reduced HFD-induced macrophage accumulation. In the liver, there was no such increase of macrophages by UTS-001 in chow fed animals, therefore the apparent increase in adipose tissue is not a general effect of UTS-001. We are not certain of the exact cause of this increase, but as UTS-001 is lipid based it could be that adipocytes partially recognise UTS-001 as a lipid and begin normal homeostatic mechanisms, i.e., recruit macrophages to adipose tissue. This dual response of lipids is common, for example, PGE2 treatment of cells results in the induction of inflammatory mediators [42] whereas PGE2 production from macrophages is an important anti-inflammatory signal to inhibit the production of pro-inflammatory cytokines [43].

Glucose intolerance is considered to be a high risk of developing type 2 diabetes [44]. FOXO-1 plays a key role in gluconeogenesis and increases blood glucose levels [45,46]. The deletion of FOXO-1 in mice has been shown to normalise glucose tolerance [45,47,48]. In the current study, glucose intolerance is increased substantially in HFD fed mice with several folds increase in FOXO-1. UTS-001 (6 mg) treatment in HFD fed mice decrease FOXO-1 expression indicating the mechanism underlying improved glucose tolerance also partially due to reduced liver gluconeogenesis. Furthermore, it has been shown that PPARγ can directly regulate the genes responsible for lipid homeostasis [49]. In response to HFD feeding, PPARγ is the early-induced lipogenic transcription factor in the liver [50,51]. FFA treatment can cause up-regulation of PPARγ via PGC1α in liver cells, and both PGC1α and PPARγ were elevated in the liver of mice with nonalcoholic fatty liver disease, a disease closely associated with obesity and HFD consumption [52]. In agreement with the literature, in the current study, HFD fed mice displayed several-fold increases in mRNA expression of liver PPARγ and PGC-1α in the face of increased liver TG concentration. Although no significant effect was seen in PGC-1α after UTS-001 (6 mg) treatment, PPARγ was significantly reduced. An increase in expression of PPARγ is one of the features of the steatotic liver and it is suggested that inhibiting the activity of PPARγ would be beneficial in the prevention of steatosis [53] and improving glucose metabolism [52]. PPAR γ is a likely target for small lipophilic compounds derived from endogenous metabolism and nutrition such as phospholipids [54,55]. Therefore, modulating hepatic PPARγ as observed in this study might be responsible for the improved glucose metabolism whilst preventing excessive liver steatosis.

IL-1β is a pro-inflammatory cytokine and is produced as an inactive protein. Biologically active IL-1β is formed through caspase-1 activation via the NOD-, LRR- and pyrin domain-containing protein 3 (NLRP3) inflammasome [56]. This pathway has been suggested to contribute to the enhanced inflammatory state, such as that observed in obesity and type 2 diabetes [57]. Furthermore, in adipose tissue, activation of NLRP3 inflammasome results in inflammation and insulin resistance [58]. Growing evidence suggests that IL-1β up-regulation contributes to the recruitment of adipose tissue macrophages and induction of additional pro-inflammatory cytokines, contributing to impaired fat-liver crosstalk thereby resulting in insulin resistance [59,60]. Although in this study IL-1β mRNA was not upregulated in HFD-fed mice, UTS-001 suppressed IL-1β mRNA in the liver with a similar trend in the fat suggesting additional anti-inflammatory properties of UTS-001. Therefore, the immunomodulatory properties of UTS-001 cannot be attributed to a single pathway. Future studies will investigate the TLR-4-MAPK/NF-KB pathway and NLRP3 inflammasome to further elucidate the full spectrum of mechanisms of action for UTS-001.

GLUT2 is the most abundant isoform of glucose transporters in the liver which is responsible for non-insulin stimulated glucose uptake [61]. Elevated hepatic GLUT2 can be observed following HFD feeding [62] that can increase FFA synthesis blockage of the insulin receptor cascade and finally insulin resistance [63]. Here, GLUT2 is upregulated in the liver in HFD-fed mice due to increased blood glucose level, which is consistent with the literature. However, UTS-001 (6 mg) treatment did not significantly downregulate GLUT2 mRNA expression, perhaps due to the need to process excess sugar influx from the HFD. Interestingly, in the serum, FFA was somewhat reduced. Such change by UTS-001 may also contribute to improved glucose clearance during IPGTT.

In this study, although we saw a small decrease in serum FFA level, TG levels in plasma were not reduced by UTS-001 treatment. It has been demonstrated that lecithin, a natural PC, can reduce lipid vacuoles in adipocyte by releasing TG, resulting in fat loss [64]. Hence, the synthetic PC in UTS-001, DOPC, might have a similar effect on adipocytes. Upregulation of ATGL in HFD-UTS-001 (6 mg) in our study could indicate increased lipolysis, resulting in TG release from the fat tissue, hence the unchanged in serum TG level and fat loss. PPARγ regulates fat cell differentiation which was unchanged by UTS-001. Our in vitro study found fat cell size although not significant, was smaller in the presence of high doses of UTS-001 although fat droplet number was not changed, suggesting the role of increased lipolysis. CPT-1a is an enzyme that regulates the transportation of long-chain fatty acid into the mitochondria for β-oxidation and has a crucial role in energy homeostasis [65]. The inhibition of CPT-1a can improve insulin sensitivity in obese mice [66] and humans [67]. Unchanged liver CPT-1a expression in HFD-UTS-001 (6 mg) may suggest that TG influx into the liver was not changed although normalised CPT-1a level in the fat may suggest that the influx into the adipose tissue may be reduced Further research is warranted to understand whether this dynamic will change in the longer term, when fat mass is normalised to the control level.

UCP-1 is a thermogenic protein located in mitochondria of brown adipose tissue and there is accumulating evidence that decreased expression of UCP-1 is linked to an increase in obesity and vice-versa [68]. In the current study, we saw a marked increase in UCP-1 mRNA expression in HFD-UTS-001 (6 mg) mice. Therefore, the increase in thermogenesis could be the other potential mechanism for fat loss.

In addition to all the mechanisms discussed above, oleic acid, resulting from DOPC metabolism in the body, has also been shown to reduce obesity in humans and demonstrate anti-inflammatory properties [69,70]. Furthermore, choline, another product of DOPC metabolism can also promote weight loss [71]. However, such effects of DOPC can only partially contribute to the overall effects of UTS-001, as shown in our supplementary results of the DOPC-liposome. Other unknown metabolic substrates from UTS-001 may play a role, which requires further investigation.

Liposomes are safe and biocompatible PC-based formulations and several liposomal formulations have been approved as drug carriers by regulatory agencies. The liposomal formulation presented in this study is an aqueous-based formulation. Several studies indicated the efficacy of nature PC in reducing obesity. For instance, Lee et al. demonstrated the prophylactic administration of PC for 12 weeks in HFD fed mice alleviated HFD induced increases in body weight, mesenteric fat mass, triglyceride and total cholesterol levels [17]. In the clinical practice, lecithin/DC injections (Lipostablil or Lipodissolve) have been used locally to decrease fat deposits [72,73,74], attributed to the lysis of adipose cells. However, such lysis is not cell-selective, thus LipoDissolve induces significant side effects of inflammatory responses and neuronal damage [64,74,75,76]. Unlike previous studies, DOPC, used in this study is a pure synthetic PC and DOPC-cholesterol liposomes (UTS-001) have shown superior efficacy in body weight management and glycaemic control compared to the components of formulation (DOPC and cholesterol mixture) (Supplementary Table S1 and Figure S1).

In conclusion, our findings demonstrate the strong efficacy of UTS-001 that can affect multiple biological pathways involved in the development of obesity and related metabolic disorders and represent a promising novel therapeutic strategy. Specific mechanisms behind improved glucose update and anti-inflammatory properties will be further elucidated in future studies. Furthermore, UTS-001 does not require further processing or functionalization to achieve its immunomodulatory properties, hence facilitating its translation to the clinic. The results presented here support the need for further investigation into the immunomodulatory properties of UTS-001 in other chronic inflammatory conditions.

## 4. Materials and Methods

### 4.1. Animal Experiments

**Figure 8:**
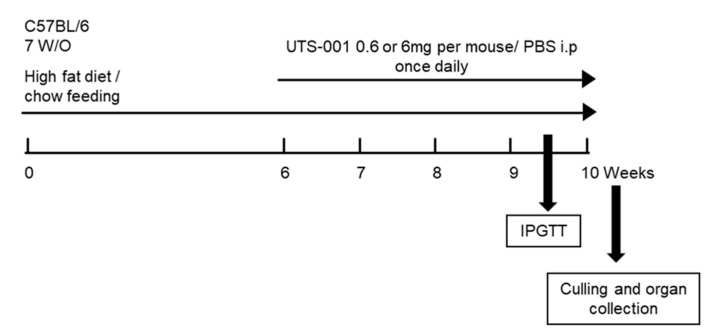
Schematic diagram of animal experiments.

Liposomes were prepared as previously described [22]. The animal experiments were approved by the Animal Care and Ethics Committee of the University of Technology Sydney (Ethics no: ETH18-2214, Approved on 15/5/2018) and carried out according to the Australian National Health and Medical Research Council Guide for the Care and Use of Laboratory Animals. Six-week-old C57BL/6 mice (male) were purchased from the Animal Resource Centre, WA, Australia and were randomized into 6 groups after a week of acclimatization. Chow group mice were fed a chow diet (12 KJ/g, 14% fat, Gordon’s Specialty Stockfeeds, NSW, Australia) and HFD group mice were fed a high-fat diet (20 KJ/g, 43% fat, Speciality feeds, Glen Forrest, WA, Australia) for 6 weeks. From the 7th week, an intraperitoneal (i.p.) injection of either (1) PBS (vehicle control); (2) 0.6 mg UTS-001/day; or (3) 6 mg UTS-001/day, was administered once daily for 4 weeks in HFD-fed mice with diet continuation (Figure 8). Body weights and 24-h caloric-intake were monitored weekly. An intraperitoneal glucose tolerance test (IPGTT) was conducted three days before the endpoint using our published method [77]. AUC was calculated using 0.5 × (Glucose_T0_ + Glucose_T15_) × (15 − 0) + 0.5 × (Glucose_T15_ + Glucose_T30_) × (30 − 15) + 0.5 × (Glucose_T30_ + Glucose_T60_) × (60 − 30) + 0.5 × (Glucose_T60_ + Glucose_T90_) × (90 − 60). Fasting blood glucose levels were measured at the endpoint using a hand-held glucose metre (Accu-Check^®^, Roche Diagnostics GmbH, Sandhofer Strasse, Mannheim, Germany) and plasma was stored at −20 °C. The heart, liver, spleen, kidneys, and fat pads were weighed. Liver and fat tissue were either fixed in 10% formalin or stored at −80°C for further analysis. All analyses were performed blindly and the results were only identified before data analysis.

#### 4.1.1. Bioassays

Plasma non-esterified free fatty acids (NEFA) were measured using a NEFA kit (WAKO, Osaka, Japan). Liver TG was extracted by the Folch method using chloroform: methanol (2:1) mixture as previously described [78]. TG was measured in plasma by an in-house assay using glycerol standards (Sigma-Aldrich, St. Louis, MO, USA) and triglyceride reagent (Roche Diagnostics, Branchburg, NJ, USA) [79]. Plasma insulin levels were measured using a Mouse Insulin ELISA kit (Abnova, Taipei, Taiwan) following the manufacturer’s instructions. The Homeostatic Model Assessment of Insulin Resistance (HOMA-IR) was calculated according to the formula: insulin (μU/mL) × glucose (mM)/22.5.

For oil red O staining, the oil red O working solution was prepared using 0.25% w/v oil red O stain (Sigma-Aldrich, Castle Hill, NSW, Australia) in isopropanol. Oil red O working solution was added to the frozen liver (30–40 mg) and homogenised. Samples were extracted with isopropanol. The optical density was measured at 520 nm and corrected by tissue weight.

#### 4.1.2. Immunohistochemistry

Formalin-fixed liver and abdominal fat samples were embedded in paraffin and sectioned (5 µm). The sections were then deparaffinized and rehydrated using xylene and decreasing grades of ethanol. Antigen retrieval was performed [80] before incubating with a rabbit anti-mouse F4/80 (1:400, Abcam, Cambridge, UK), rabbit anti-mouse CD68 (1:300, Abcam, Cambridge, UK) primary antibodies. Horseradish peroxidase anti-rabbit Envision system (Dako Cytochemistry, Tokyo, Japan) was used to visualize. The sections were then counterstained with hematoxylin. Three images from each section were captured and used for analysis with ImageJ (National Institutes of Health, Bethesda, MD, USA).

#### 4.1.3. Quantitative Real-Time PCR

Total RNA was isolated from the tissues using Trizol reagent (Sigma-Aldrich, St. Louis, MO, USA). First-strand cDNA was synthesized using M-MLV Reverse Transcriptase, RNase H Minus, Point Mutant Kit (Promega, Fitchburg, WI, USA). Pre-optimized TaqMan^®^ probe/primer and SYBR green expression assays primers (information in Supplementary Tables S2 and S3, Life Technologies, Carlsbad, CA, USA) were used for the real-time PCR (Eppendorf Realplex2, Hamburg, Germany). The genes of interest were normalised against the 18s rRNA. The average value of the Chow-PBS was set as the calibrator, against which all other samples are expressed as a fold difference.

### 4.2. Cell Culture Studies and Cellular 2-NBDG Uptake

The Murine 3T3-L1 fibroblasts were purchased from Cell bank Australia, NSW, Australia and were maintained at 5% CO_2_ and 37 °C. Murine 3T3-L1 fibroblasts were differentiated into mature adipocytes using a previously published protocol [81,82].

To measure the uptake of glucose into adipocytes, 2-NBDG uptake was used. Mature adipocytes were pretreated with PBS, Metformin (1 μM), or UTS-001 (0.375, 1.125, 2.25, 3.375 mg of lipids/mL) in high glucose DMEM for 24 h. Following incubation, the adipocytes were washed with PBS and serum-starved for 3 h using 0.1% BSA in glucose-free phenol red free DMEM. Adipocytes were then incubated with 160 μM of 2-NBDG (2-(N-(7-Nitrobenz-2-oxa-1,3-diazol-4-yl)Amino)-2-Deoxyglucose) and insulin (100 nM) for 60 min in serum and glucose-free DMEM. The 2-NBDG uptake was ceased by washing the cells with precooled PBS. The fluorescence was measured by a Tecan microplate reader at an excitation wavelength of 485 nm and an emission wavelength of 535 nm.

In order to measure the diameter of the lipid droplets following the treatment with UTS-001, Oil Red O staining was used. In brief, on day 8, mature adipocytes were treated with PBS, or UTS-001 (0.375, 1.125, 2.25, 3.375 mg of lipids/mL) for 48 h. Following incubation, the adipocytes were washed with PBS and then fixed with 4% ice cold paraformaldehyde for 30 min followed by washing with 60% isopropyl alcohol. Fixed cells were then stained with 0.1% Oil Red O working solution for 15 min and then rinsed with milli-Q water. Dapi (300 nM) was used to counterstain the cells. The cells were imaged with IN Cell Analyser 2,200 (GE Healthcare, Issaquah, WA, USA) and the images were analysed by ImageJ software.

### 4.3. Statistical Analysis

Data are represented as mean ± S.E.M. Data were analyzed using two-way ANOVA, followed by post hoc uncorrected Fisher’s LSD tests if the data is normally distributed. If the data were not normally distributed, they were log-transformed to achieve normality of distribution before analysis. (Graphpad Prism software, San Diego, CA, USA). For in vitro studies and HFD-fed mice treated with individual components, data were analyzed using one-way ANOVA, followed by post hoc uncorrected Fisher’s LSD tests.

## Figures and Tables

**Table 1:** Effects on anthropometric parameters of mice with HFD and UTS-001 treatment.

Parameters	Chow-PBS	Chow-UTS-001 (0.6 mg)	Chow-UTS-001 (6 mg)	HFD-PBS	HFD-UTS-001 (0.6 mg)	HFD-UTS-001 (6 mg)
Body weight at 0 week (g)	19.9 ± 0.3	20.3 ± 0.1	19.8 ± 0.3	19.5 ± 0.3	20.1 ± 0.3	19.9 ± 0.3
Body weight at 10 weeks (g)	28.7 ± 0.5	29.5 ± 0.5	28.1 ± 0.4	37.1 ± 0.8 ****	38.3 ± 1.5 ****	33.8 ± 1.2 ****^,##^
Twenty-four calorie intake (kJ/mouse/day)	36.18 ± 2.18	28.05 ± 0.69	37.45 ± 3.25	238.4 ± 33.13 ****	318.53 ± 37.27 ****^#^	261.41 ± 30.06 ****
Kidney (g)	0.192 ± 0.005	0.193 ± 0.012	0.181 ± 0.009	0.215 ± 0.007 *	0.213 ± 0.014	0.213 ± 0.009
%	0.690 ± 0.018	0.690 ± 0.018	0.670 ± 0.031	0.603 ± 0.027 *	0.592 ± 0.021 *	0.642 ± 0.019
Liver (g)	1.18 ± 0.005	1.27 ± 0.03	1.22 ± 0.04	1.56 ± 0.07 ****	1.58 ± 0.07 **	1.400 ± 0.040 **^,#^
%	4.23 ± 0.15	0.683 ± 0.037	4.51 ± 0.12	4.30 ± 0.12	4.46 ± 0.31	4.23 ± 0.11
Heart (g)	0.143 ± 0.005	0.146 ± 0.007	0.139 ± 0.008	0.159 ± 0.006	0.150 ± 0.008	0.149 ± 0.012
%	0.511 ± 0.012	0.514 ± 0.018	0.517 ± 0.030	0.433 ± 0.015 *	0.422 ± 0.019 **	0.433 ± 0.018 *
Spleen (g)	0.096 ± 0.012	0.087 ± 0.007	0.085 ± 0.014	0.110 ± 0.007	0.096 ± 0.015	0.105 ± 0.011
%	0.343 ± 0.041	0.308 ± 0.021	0.312 ± 0.042	0.298 ± 0.014	0.259 ± 0.029	0.305 ± 0.020
Rp fat (g)	0.091 ± 0.010	0.107 ± 0.012	0.077 ± 0.013	0.680 ± 0.057 ****	0.767 ± 0.111 ****	0.405 ± 0.070 ***^,##^
%	0.324 ± 0.033	0.380 ± 0.040	0.284 ± 0.046	1.86 ± 0.12 ****	2.17 ± 0.36 ****	1.19 ± 0.18 ****^,##^
Testicular fat (g)	0.381 ± 0.017	0.444 ± 0.044	0.312 ± 0.034	1.81 ± 0.13 ****	1.93 ± 0.15 ****	1.04 ± 0.16 ****^,####^
%	1.37 ± 0.06	1.57 ± 0.15	1.16 ± 0.12	4.95 ± 0.29 ****	5.36 ± 0.30 ****	3.05 ± 0.40 ****^,####^

Data are expressed as mean ± S.E.M. and were analysed using two-way ANOVA, followed by post hoc Fisher’s LSD tests. *n* = 6–12. * *p* < 0.05 vs. Chow-PBS; ** *p* < 0.01 vs. Chow-PBS; *** *p* < 0.001 vs. Chow-PBS; **** *p* < 0.0001 vs. Chow-PBS, # *p* < 0.05 vs. HFD-PBS; ## *p* < 0.01 vs. HFD-PBS; ### *p* < 0.001 vs. HFD-PBS; #### *p* < 0.0001 vs. HFD-PBS.

**Table 2:** Lipid and glucose profiles in mice with HFD and UTS-001 treatment.

Parameters	Chow-PBS	Chow-UTS-001 (0.6 mg)	Chow-UTS-001 (6 mg)	HFD-PBS	HFD-UTS-001 (0.6 mg)	HFD-UTS-001 (6 mg)
Serum TG (mg/mL)	0.94 ± 0.032	1.15 ± 0.05	1.06 ± 0.047	1.12 ± 0.047*	1.21 ± 0.062 **	1.19 ± 0.062 ***
Serum FFA (nM)	4.82 ± 0.21	4.74 ± 0.27	4.9 ± 0.25	5.49 ± 0.35	5.95 ± 0.48	4.8 ± 0.15
Liver Oil Red O staining (OD/mg of tissue)	0.0057 ± 0.00032	0.0066 ± 0.00059	0.00501 ± 0.00031	0.01898 ± 0.00321 ***	0.02489 ± 0.0016 ****	0.019 ± 0.00346 ****
Glucose (mM)	12.8 ± 1.2	12.9 ± 1.4	14.8 ± 0.3	17.5 ± 1.2 **	19.15 ± 0.9 ***	18.1 ± 1.1 **
Serum insulin (ng/mL)	0.399 ± 0.02	0.40 ± 0.04	0.409 ± 0.04	0.412 ± 0.02	0.399 ± 0.03	0.415 ± 0.03
HOMA-IR	0.22 ± 0.02	0.24 ± 0.03	0.27 ± 0.01	0.34 ± 0.02 ****	0.34 ± 0.01 ***	0.32 ± 0.02 ***

Data are expressed as mean ± S.E.M. and were analysed using two-way ANOVA, followed by post hoc Fisher’s LSD tests. *n* = 7–12. * *p* < 0.05 vs. Chow-PBS; ** *p* < 0.01 vs. Chow-PBS; *** *p* < 0.001 vs. Chow-PBS; **** *p* < 0.0001 vs. Chow-PBS.

## Supplementary Materials

Supplementary materials can be found at https://www.mdpi.com/1422-0067/21/20/7640/s1.

## Abbreviations

IL	Interleukin
FASN	Fatty acid synthase
SREBP-1c	Sterol regulatory element-binding protein 1
MCP-1	Monocyte chemoattractant protein-1
FOXO-1	Forkhead box protein O1
ATGL	Adipose triglyceride lipase
TNF-α	Tumor necrosis factor-alpha
UCP-1	Uncoupling protein-1
UCP-3	Uncoupling protein-3
PPARγ	Peroxisome Proliferator Activated Receptor Gamma
TLR4	Toll-like receptor-4
CPT1a	Carnitine palmitoyltransferase 1a
PGC1 α	Peroxisome proliferator-activated receptor gamma coactivator 1-α
GLUT 2	Glucose transporter 2
DOPC	1,2-dioleoyl-sn-glycero-3-phosphocholine
NLRP3	NOD-, LRR- and pyrin domain-containing protein 3
