# Supplementary material for: Impact of A Cargo-Less Liposomal Formulation on Dietary Obesity-Related Metabolic Disorders in Mice
Source: Int J Mol Sci. 2020 Oct 15;21(20):7640. doi: 10.3390/ijms21207640 (PMC7589567; doi:10.3390/ijms21207640)
Supplement: Supplementary file 1 [file ijms-21-07640-s001.pdf]

Impact of Cargo-less Liposomal Formulation on Dietary Obesity-Related Metabolic Disorders in Mice

Varsha Komalla[1,2], Behjat Sheikholeslami[1,2], Gerard Li[3], Bishwajit Bokshi[1,4], Yik Lung Chan[2,3], Alison Ung[4], Brian Gregory Oliver[2,3], Hui Chen[3], Mehra Haghi[1*]

1 Graduate School of Health, University of Technology Sydney, Chippendale, NSW 2008, Australia
2 Woolcock Institute of Medical Research, The University of Sydney, Glebe, NSW 2037, Australia
3 School of Life Sciences, University of Technology Sydney, Ultimo, NSW 2007, Australia
4 School of Mathematical and Physical Sciences, University of Technology Sydney, Ultimo, NSW 2007, Australia

* Correspondence: Mehra Haghi, Graduate School of Health, University of Technology Sydney, Building 20, 100-102 Broadway, Chippendale, NSW 2008, Australia. E-mail: Mehra.Haghi@uts.edu.au. Phone: +61 2 95149296.

Supplementary Methods:
The animal experiments were approved by the Animal Care and Ethics Committee of the University of Technology Sydney (Ethics no: ETH18-2214) and carried out according to the Australian National Health and Medical Research Council Guide for the Care and Use of Laboratory Animals. Six-week-old C57BL/6 mice (male, Australian Resource Centre, WA, Australia) were randomized into 3 groups after a week of acclimatisation. The high-fat diet (HFD) group mice were fed a pellete HFD (20 KJ/g, 43% fat, Speciality feeds, WA, Australia) for 6 weeks. From the 7th week, an intraperitoneal (i.p) injection of either 1) Corn oil (vehicle control), 2) 1,2-dioleoyl-sn-glycero-3-phosphocholine (DOPC) in the form of liposomes, or 3) DOPC + Cholesterol in corn oil, was administered once daily for a total of 4 weeks in HFD-fed mice with the HFD feeding. Body weights were monitored weekly. Intraperitoneal glucose tolerance test (IPGTT) was performed three days before the endpoint. Fat pads were collected and weighed.

Results

DOPC liposome treatment induced a 5% reduction in body weight compared with HFD-oil mice, with significantly reduced fat mass ($P<0.05$ for retroperitoneal (Rp) fat, $P<0.01$ for testicular fat vs HFD-Oil group, Table A1). DOPC + cholesterol mixture had no impact on body weight, nor fat mass.

As shown in Figure A1, HFD-fed mice receiving DOPC liposomes treatment had some improvement in blood glucose levels during IPGTT in comparison with the HFD-Oil group ($P<0.001$ for 60 and 90 mins). DOPC + cholesterol mixture only reduced blood glucose level at 60 min during IPGTT ($P<0.001$ vs HFD-oil). The area under the curve (AUC) was significantly lower in the HFD-DOPC liposome group ($P<0.05$ compared to the HFD-Oil group). Interestingly, no significant difference was seen between HFD-DOPC+Cholesterol and HFD-oil groups.

Appendix A

Table 1. Anthropometric parameters of the mice treated with oil, DOPC liposomes, and DOPC+Cholesterol mixture.

Parameters	HFD-Oil	HFD-DOPC liposomes	HFD-DOPC+cholesterol
Body weight at 0 week (g)	18.73 ± 0.30	18.98 ± 0.36	18.98 ± 0.26
Body weight at 10 weeks (g)	36.35 ± 1.30	34.47 ± 1.12	36.28 ± 0.91
Rp fat (g)	0.69 ± 0.06	0.46 ± 0.07*	0.58 ± 0.07
%	1.87 ± 0.17	1.30 ± 0.18**	1.57 ± 0.17
Testicular fat (g)	1.70 ± 0.14	1.16 ± 0.19**	1.46 ± 0.18
%	4.63 ± 0.25	3.31 ± 0.46**	3.98 ± 0.44

Data are expressed as mean ± S.E.M. and were analysed using one-way ANOVA, followed by post hoc Fisher's LSD tests. n = 12. * P < 0.05 vs. HFD-Oil; ** P < 0.01 vs. HFD-Oil.

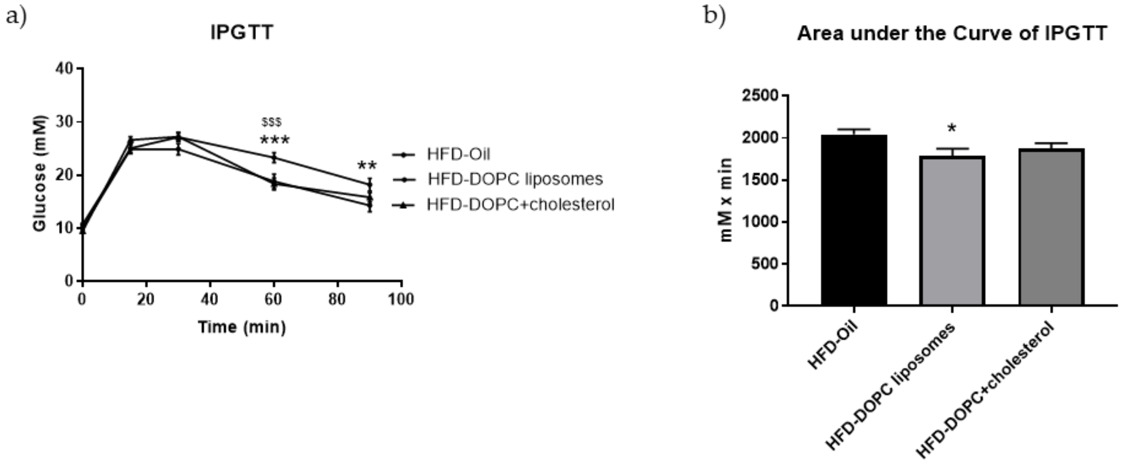

Figure 1. a) An intraperitoneal glucose tolerance test (IPGTT, glucose 2g/kg) after 4 weeks of treatments. b) Area under the curve (AUC) of the IPGTT in (a). Results are expressed as mean ± S.E.M, n = 10-12. a) Data were analyzed using two-way ANOVA followed by post hoc Fischer's LSD test. b) Data were analysed using one-way ANOVA by post hoc Fischer's LSD test. *** P < 0.001, HFD-oil vs.HFD-DOPC liposomes at 60 mins; $$$P<0.001, HFD-oil vs. HFD-DOPC+Cholesterol at 60 mins;. ** P < 0.01, HFD-oil vs.HFD-DOPC liposomes at 90 mins.

Table A2. TaqMan® probe information (Life Technologies, CA, USA)

Gene	NCBI gene references	Probe sequence	Assay ID
TNFα	NM_013693.2,X02611.1,M13049.1	CCCTCACACTCAGATCATCTTCT CA	Mm00443259_g1
TLR4	NM_021297.2,AF095353.1,AF110133.1	CCCTGCATAGAGGTAGTTCCTAA TA	Mm00445273_m1
FASN	NM_007988.3,AF127033.1,AK147214.1	AGCAATTGTGGATGGAGGTATCA AC	Mm00662319_m1
PPAR-γ	NM_0011273330.1	ATGCTGTTATGGGTGAAACTCTG G	Mm01184322_m1
Foxo1	NM_019739.3,AK154041.1,AF126056.1	TCGGCGGGCTGGAAGAATTCAAT TC	Mm00490671_m1
Cpt1a	NM_013495.2,AK147770.1,AK136487.1	TTCCAGGAGAATGCCAGGAGGT CAT	Mm01231183_m1
Ppargc1a	NR_027710.1,NM_008904.2,JX866947.1	CTGGAACTGCAGGCCTAACTCCT CC	Mm01208835_m1
IL-6	NM_031168.1,X06203.1,X54542.1	ATGAGAAAAGAGTTGTGCAATG GCA	Mm00446190_m1
GLUT2	NM_031197.2	CCGCCTCCCCCGGCGCGCACACA CC	Mm00446229_m1
Ucp1	NM_009463.3,U63419.1,AK002759.1	TTTCAAAGGGTTTGTGGCTTCTTT T	Mm01244861_m1
Ucp3	NM_009464.3,AF032902.1,AF030164.1	GTGGAAAGGGACTTGGCCCAAC ATC	Mm01163394_m1
ATGL	NM_025802.3	CCAAGACTGAATGGCTGGATGG CAA	Mm00503040_m1
MCP-1	NM_011333.3	TCAGCCAGATGCAGTTAACGCCC CA	Mm00441242_m1

Table 3. SYBR® primer information (Sigma-Aldrich, MO, USA).

Gene	Forward primer sequence (5' → 3')	Reverse primer sequence (5' → 3')
SREBP-1c	AATAAATCTGCTGTCTTGCG	CCTTCAGTGATTTGCTTTTG
